# A study on canine dirofilariasis in selected areas of Sri Lanka

**DOI:** 10.1186/s13104-022-06024-0

**Published:** 2022-04-14

**Authors:** Randima Dasanayake, Thivya Balendran, Dhilma Atapattu, Devika Iddawela

**Affiliations:** 1 Animal Quarantine Office, Bandaranayake International Airport, Katunayake, Sri Lanka; 2 Department of Parasitology, Faculty of Medicine, University of Peradeniya, Peradeniya, Sri Lanka

**Keywords:** Canine dirofilariasis, *Dirofilaria repens*, *Dirofilaria immitis*, Mongrels, Sri Lanka

## Abstract

**Objectives:**

Canine dirofilariasis is a mosquito borne zoonotic filarial parasitic disease caused by *Dirofilaria* species. In Sri Lanka, human dirofilariasis is endemic and well documented. Even though canine dirofilariasis is an established infection among dog population**s** in Sri Lanka, the prevalence and risk factors were not documented in Kanthale divisional secretariat, Eastern Province of Sri Lanka. Therefore, the main objective of this study is to determine the prevalence of dirofilariasis and to identify the exposure-related risk factors in dogs using an interviewer administered questionnaire in Kanthale divisional secretariat.

**Results:**

Out of 162 blood samples collected from dogs, 47.5% (n = 77/162) were positive for the presence of microfilariae by direct smear. Of 162 samples 58.6% (n = 95/162) were molecularly identified as *D. repens*. Species-specific primer pair DIR3/DIR4 that amplified 5S rRNA was used. The current study observed a significant association between mongrels and canine dirofilariasis (*p* = 0.049), where 61.3% (n = 95) out of 155 mongrels showed PCR positivity. This study showed no significant association between the occurrence of dirofilariasis and the age, sex, rearing method, presence or absence of skin rash, and stray or domestic dogs. *Dirofilaria immitis* was not identified in direct smear test and PCR.

**Supplementary Information:**

The online version contains supplementary material available at 10.1186/s13104-022-06024-0.

## Introduction

Canine dirofilariasis is a mosquito borne zoonotic filarial parasitic disease caused by *Dirofilaria* species. *Dirofilaria immiitis* and autochtonous *Dirofilaria repens* are the etiological agents responsible for pulmonary dirofilariasis and subcutaneous dirofilarisis, respectively, in dogs [1]. *Dirofilaria repens* infection in dogs is limited to Asia, Africa and Europe [2]. Higher prevalence of *D. repens* is reported in Spain (84.6%) [3], France (35.2%) [4], and Greece (30%) [3]. Prevalence of *D. repens* reported in Sri Lanka ranged between 30% and 60% [5–7]. *Dirofilaria immitis* was not reported in Sri Lanka yet.

Age, sex, breed, climate, size, provenance, management system, and pollution source, can be potential risk factors for canine dirofilariasis [1, 8]. The common clinical presentation of *D. repens* infection includes subcutaneous nodules, subconjuctival lesions and meningo encephalitis in dogs [9]. Progression of *D. repens* in dogs is mainly asymptomatic causing silent spread of infection among dogs [8]. Thus, eventually increases human infection [10]. Human acts as an accidental host for this infection [11]. In humans, it presents as localized nodules in skin, sub conjunctival, or peri-orbital tissues [6, 12].

Even though this infection is known for a period of 80 years, only few studies have been carried out on canine dirofilariasis [6]. A study carried out in Sri Lanka has shown an increased dog population (ratio of 1:4.6 dog to human population) [13]. Therefore, this study was designed to detect the prevalence of canine dirofilariasis and to identify the risk factors of *Dirofilaria* infection in dogs in Kanthale divisional secretariat in Trincomalee district in Sri Lanka.

## Main text

### Method

#### Study area

The study was conducted in Kanthale divisional secretariat, Trincomalee district, located in the Eastern Province of Sri Lanka. Blood samples were collected from the dogs attending Anti-rabies vaccination program from four village officers (VO) divisions. The VO divisions were randomly selected. Dog population was determined according to the Department of Animal Production and Health calculation with a dog to human population ratio of 1:6 and calculated dog population in the study area was 11,000 and confidence interval (CI) was 95%.

#### Sample collection

A total of 162 samples were collected from stray and domestic dogs, which were brought to the anti-rabies vaccination programs in the study area. All dogs aged above 6 months were included in this study. Blood samples were collected by the veterinary surgeon from the cephalic vein using sterile disposable syringe into EDTA tubes under strict aseptic conditions. The samples were stored at 4 ºC until dispatched to the Department of Parasitology, Faculty of Medicine, and University of Peradeniya. Demographical data and noduleswere recorded using an interviewer administered questionnaire. The questionnaires were filled by the author.

#### Detection of microfilariae (mf)

##### Thick blood smear

Thick blood smear was carried out according to the method described by Phuakrod et al. [14]. The smears were incubated at 37 °C overnight. The samples were hemolyzed with clean water and fixed with methanol for 30 s and fixed samples were covered with 5–10 drops of Giemsa stain (1:10 dilution) for 10–15 min and excess stain was washed with running tap water and air dried. The stained smears were observed under light microscope in higher magnification (× 100 objective lens). Microfilariae were identified based on the morphological key published by Mallawarachchi et al. [15] and Liotta et al. [16].

#### DNA extraction

Genomic DNA was extracted from all blood samples using commercially available DNA extraction kit (Gene JET Genomic DNA Purification Kit) according to the manufacturer’s guidelines.

#### Amplification of *Dirofilaria species*

PCR was done for all 162 samples using *D. immitis* and *D. repens* specific primers separately (Table 1). Tenfold dilution of extracted DNA was used to carry out PCR to identify 13 mf positive but PCR negative samples.

**Table 1:** Primers used for *Dirofilaria* species identification

Primer pair	Primer sequence	Gene target	Product origin	Product size (base-pairs)	Reference
*D.imm-*F1	CATCAGGTGATGATGTGATGAT	ITS 2	*D. immitis*	302	[17]
*D.imm-*R1	TGATTGGATTTTAACGTATCATT				
*DIR 3*	CCGGTAGACCATGGCATTAT	5S rRNA	*D. repens*	246	[18]
*DIR 4*	CGGTCTTGGACGTTTGGTTA				

*ITS* internal transcribed spacer, *5S rRNA* ribosomal 5S ribonucleic acid.

Amplification for *D. immitis* was carried out in 25 μl reaction mixture composed of 2.5 μl of PCR buffer, 2 μl of 2.5 mM dNTP, 1.5 μl of forward and reverse primer (10 pmol), 0.25 μl of Taq DNA polymerase, 4 μl of 25 mM MgCl_2_, 5 μl of template DNA and 8.25 μl of nuclease free water. The temperature profile was initial denaturation at 94 ºC for 2 min followed by 32 cycles each at 94 ºC for 30 s, the annealing 60 ºC for 30 s, 72 ºC for 1 min with final extension at 72 ºC for 5 min [19]. The temperature profile for *D. repens* amplification was initial denaturation at 94 ºC for 3 min followed by 40 cycles each at 94 ºC for 30 s, the annealing at 55 ºC for 30 s, 72 ºC for 1 min and the final extension was at 72 ºC for 5 min [20]. Amplified products were analyzed by agarose gel electrophoresis. All raw gel images are given in Additional file 1.

#### Statistical analysis

Descriptive statistical analysis was used to identify the relationship between the occurrence of canine dirofilariasis and breed, rearing method, stray vs. domestic, skin rash, age and gender. The statistical significance of relationships among selected variables and mf positivity was determined using Chi square test and the Fishers exact test. Statistical analysis was carried out by GraphPad Prism 9.1.0 (221) [21]. p < 0.05 was considered statistically significant.

## Results

### Demography of study population

A total of 162 blood samples were collected from four VO division; Sooriyapura (22, 14%), Wanela (28, 17%), Agbopura (62, 38%), and Thalgaswewa (50, 31%). Of the total of 162 samples the majority (n = 103, 64.6%) were males. Large proportion (n = 140, 86.4%) of the study group were aged above one year. Of the study population 17 (10.5%) were stray dogs and 145 (89.5%) were domestic dogs. Majority were reared outdoor (n = 127, 78.4%). Large number of dogs in the study population were mongrel (n = 155, 95.7%) (Table 2).

**Table 2:** PCR positivity by demographic and other factors of the study population

Demographic factors	PCR positive	Total	p-value
Gender			
Male	65 (63.1%)	103 (64.6%)	0.349
Female	30 (50.8%)	59 (36.4%)	
Source			
Stray	17 (100%)	17 (10.5%)	0.126
Domestic	78 (53.8%)	145 (89.5%)	
Skin rash			
Having rash	40 (48.6%)	74 (45.7%)	0.605
No rash	55 (62.5%)	88 (54.3%)	
Breed			
Mongrel	95 (61.3%)	155 (95.7%)	0.049
Other	0	7 (4.3%)	
Age			
6 months < 1 year	10 (45.4%)	22 (13.6%)	0.560
> 1 year	85 (55.7%)	140 (86.4%)	
Mode of rearing			
Indoor	23 (65.7%)	35 (21.6%)	0.645
Outdoor	72 (51.2%)	127 (78.4%)	

### Microfilariae (mf) positivity

Examination of direct smear revealed 77 (47.5%) unsheathed mf out of 162 samples. Sixty-four mf samples had the morphological features of *D. repens* with cephalic end obtuse, two of cephalic nuclei, sharp tail, and a filiform with an umbrella handing, with a width ranging from 6 to 8 μm and length ranging from 310 to 355 μm. Rest of the mf (n = 13/77) were slightly different, where they had a blunt anterior end and unsheathed posteriror ending with button hook.

### Molecular identification

**Fig. 1:**
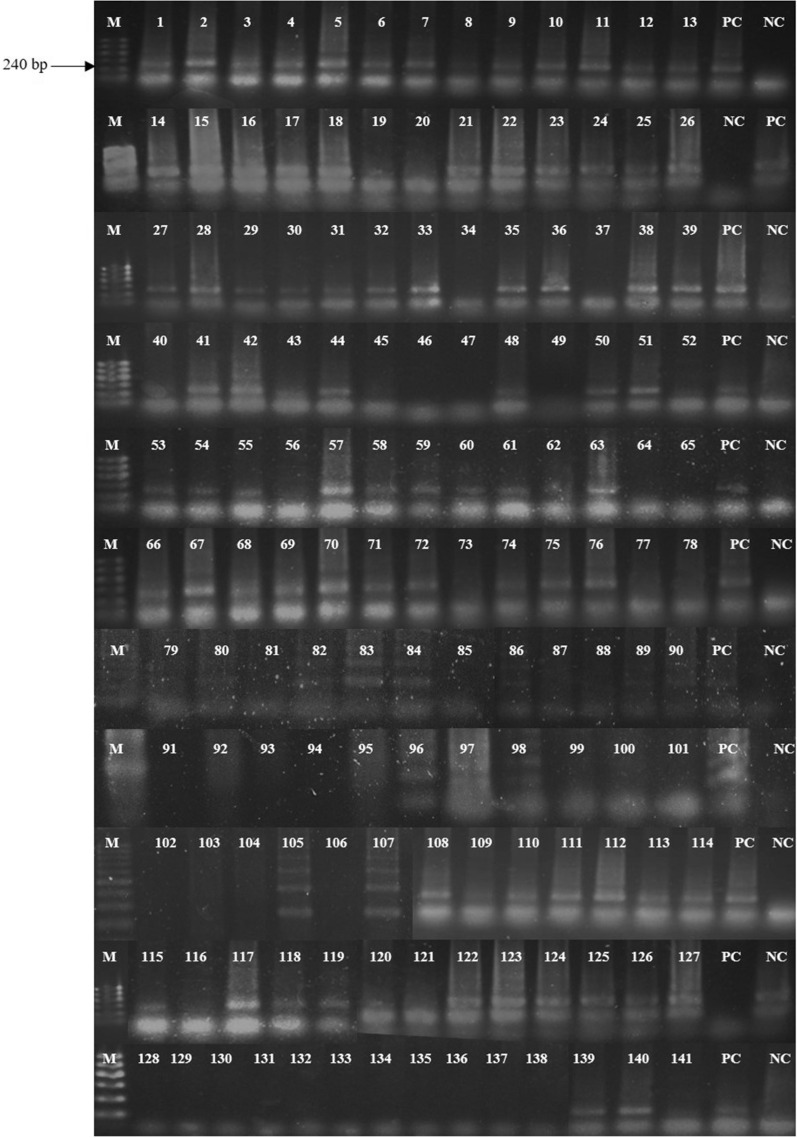
Amplification of *D. repens 5s rRNA* gene using species-specific primers DIR 3 and DIR 4. *M* molecular marker with 100 bp DNA ladder, *PC* positive control, *NC* negative control. Samples 1–18, 21–33, 35–36, 38–44, 48, 50–55, 57–63, 66–72, 74–76, 80, 82–84, 86, 89–90, 96–98, 105, 107–114, 115, 117–119, 122–127, 139–140 were PCR positive

Sixty-four microfilariae samples with morphological features of *D. repens* and 31 samples out of 85 smear negative samples were positive for *D. repens* species-specific primers, where the rest of the 13 mf samples were PCR negative for both onefold and tenfold dilution (Fig. 1). None were positive for *D. immitis* species-specific primers.

### Factors associated with dirofilaria infection

All PCR positives were considered *D. repens* infected dogs (n = 95, 58.6%). Compared to female dogs (30, 50.8%), males (65, 63.1%) showed higher *D. repens* infection. Higher percentage of stray (n = 17/17, 100.0%) were infected with *Dirofilaria* infection. Dogs aged more than 1 years showed high rate of infection (n = 85/140, 55.7%) in comparison to dogs aged above 6 months to less than 1 year (n = 10/22, 45.4%). With regard to type of rearing, outdoor reared dogs were highly infected (Table 2). Out of 95 *Dirofilaria* positive 40 (48.6%) were presented with skin rashes. There was a statistically significant difference (*p* = 0.049) of PCR positivity and the breed of the dog, showing high percentage (61.3%) of PCR positivity in mongrels (Table 2).

## Discussion

Dirofilariasis is an established infection among dogs in Sri Lanka and the main causative parasite is known to be *D. repens* [6]. Microfilaremic dogs present in an area can increase the risk of transmitting the infection to humans through competent mosquito vectors [11]. Studies conducted several decades ago revealed 30–60% prevalence of infection in dogs in some areas of Sri Lanka [6]. In the present study, the overall prevalence of 58.6% of canine dirofilariasis by *D. repens* was recorded in Kanthale divisional secretariat which includes 4 VO divisions.

Similarly, previous Sri Lankan studies reported prevalence between 44% and 54.4% in Western and North Western Provinces [6]. In contrast to present study low prevalence has been reported in India (26.5%), USA and Canada (12%) [1, 22]. Current study did not identify *D. immitis.* The only case of *D. immitis* that was reported in the country was in a dog imported from China [23].

In this study, majority of the infected dogs were males (63.1%) though it is not statistically significant (p > 0.05). Several studies have documented the similar findings [24, 25]. The current study identified a significant association of mongrels with canine dirofilariasis (*p* = 0.049), similar to the previous studies; that of mongrels being more prone to *D. repens* infection [15, 25].

The present study failed to identify statistically significant difference between age groups and the infection. But, the accumulation of infection transmission increases with age [26]. However, the majority of infected dogs (n = 85/95, 89.5%) were older than one year. Similar results were documented in several other studies [24–29]. Even though the mode of rearing and dirofilarial infection did not show a significant association, the current study observed high rate of infection in the outdoor reared dogs. A previous study has showed a significantly high infection in outdoor reared dogs [29]. In contrast to a previous study [30], the presence of skin rash with dirofilariasis infection did not show any significant association.

The results did not show any significant association with dogs being stray or domestic. The majority of the dogs having the infection were stray dogs (100.0%). Almost all the stray dogs had not been treated with anti-parasitic drugs before and this may have been the reason for the high occurrence of dirofilariasis among stray dogs. Further, studies are required to identify the factors affecting canine dirofilariasis among domestic dogs in Sri Lanka.

Out of the 77 (47.5%) microfilariae positive samples, 64 (83.1%) and 31 out of 85 smear negative samples were identified as *D. repens* by PCR. Thirteen microfilariae positive samples did not belong either to *D. repens* or *D. immitis.* There are several other *Dirofilaria* species causing canine dirofilariasis [30, 31]. Therefore, further studies are needed to identify these 13 mf positive samples. In Sri Lanka, the dirofilariasis infection in humans is on the rise [32, 33] and the local studies have identified lack of public awareness on this illness [34, 35].

In conclusion this study showed high prevalence of canine dirofilariasis due *D. repens* among dogs and mf of *D. immitis* were not detected in Kanthale division in consistent with previous reports. Further, this study identified the breed of the dog as a significant risk factor for dirofilarial infection. The current study recommends to enhance prophylaxis to prevent *Dirofilaria* species infection, treatment of infective dogs, increased public awareness, responsible dog ownership, and vector control programs in Sri Lanka in order to reduce the human and canine exposure to this zoonotic infection in Sri Lanka. Further studies are needed to further characterize dirofilarial species.

## Limitations

The sample size was limited and could not use the universal primers for Cox 1 or 12S. Sequencing was not able to carryout to identify 13 unknown microfilariae.

## Supplementary Information

**Additional file 1.** Original unprocessed gel images.

## Data Availability

The datasets used and/or analyzed during the current study are available within the manuscript and Additional file.

## Abbreviations

PCR: Polymerase chain reaction
5S rRNA: Ribosomal 5S ribonucleic acid
VO: Village officers
CI: Confidence interval
DNA: Deoxyribonucleic acid
ITS: Internal transcribed spacer
DNTP: Deoxyribonucleotide triphosphate
Bp: Base pair
MF: Microfilariae
