# Supplementary material for: A study on canine dirofilariasis in selected areas of Sri Lanka
Source: BMC Res Notes. 2022 Apr 14;15:137. doi: 10.1186/s13104-022-06024-0 (PMC9009037; doi:10.1186/s13104-022-06024-0)
Supplement: Supplementary file 1 — Additional file 1. Original unprocessed gel images. [file 13104_2022_6024_MOESM1_ESM.pdf]

4.3.2 PCR identification of *D. repens* by using *DIR 3, DIR 4* primer pair.

The microscopically positive samples (N=77) were used to do PCR using a *DIR 3, DIR 4* primer pair. Out of the 77 samples, 64 samples were positive while 13 samples were negative . Those positive samples showed bands with fragment size of 246 bp (in between 200 – 300 bp).

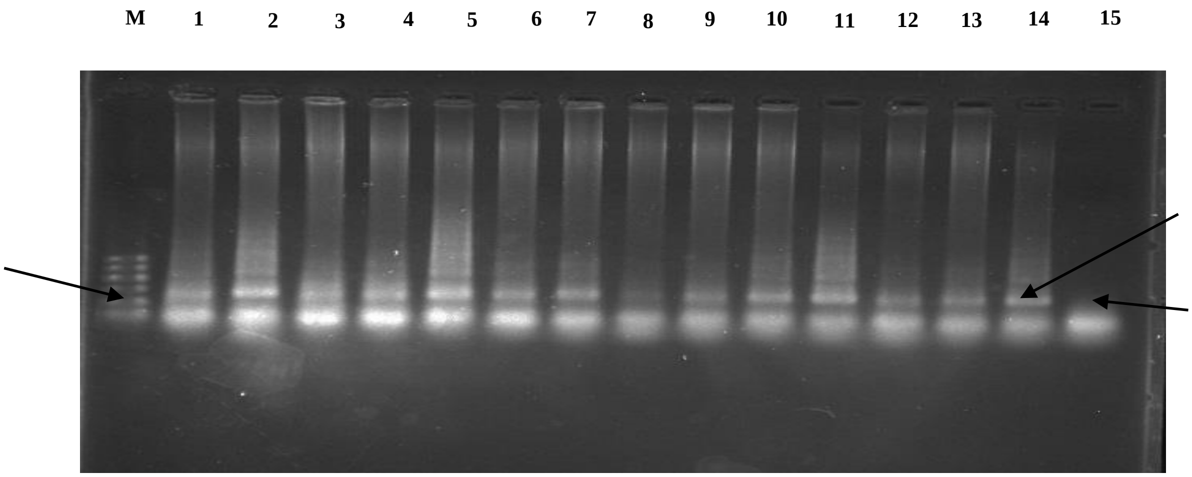

Figure 1 Gel electrophoresis photo of PCR done using *DIR 3, DIR 4* primers. M: Molecular marker with 100 bp DNA ladder, Lane 14 – Positive control, Lane 15- Negative control. Positive samples – Lane 1,2,3,4,5,6,7,8,9,10,11,12,13

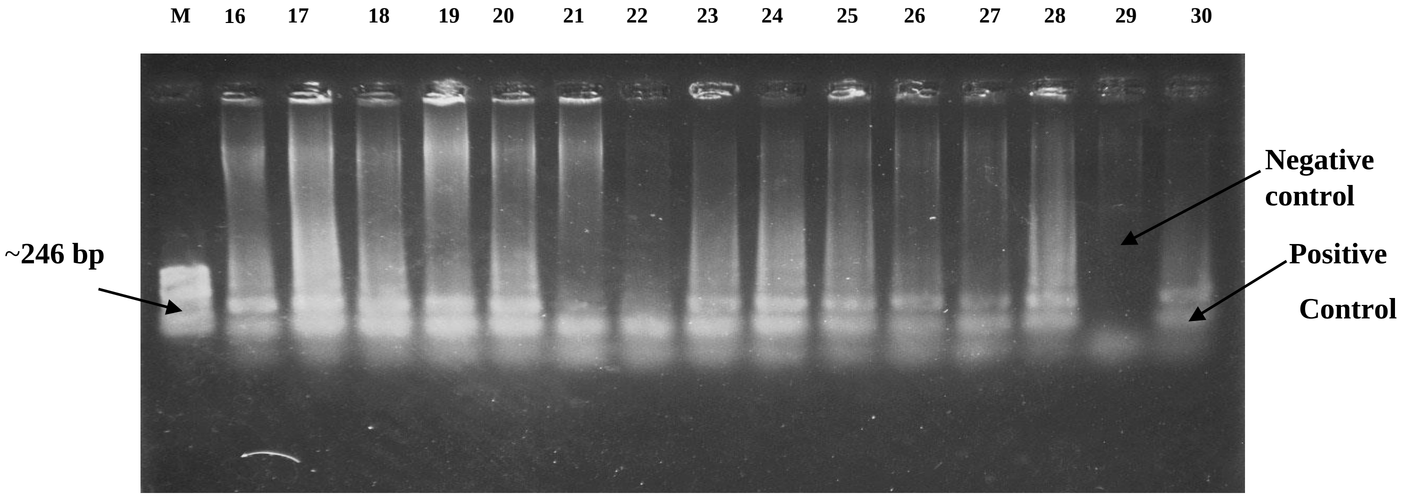

Figure 2 Gel electrophoresis photo of PCR done using*DIR 3, DIR 4* primers. M: Molecular marker with 100 bp DNA ladder, Lane 29 – Negative control, Lane 30- Positive control. Positive samples – Lane 16, 17, 18, 19, 20, 23, 24, 25, 26, 27, 28. Negative samples – Lane 21, 22.

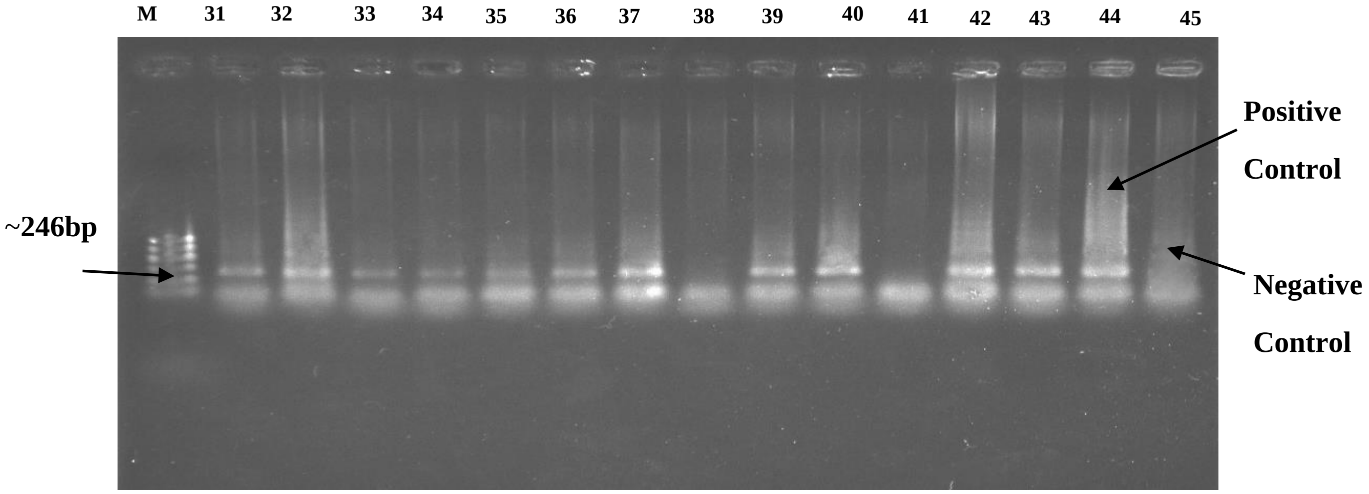

Figure 3 Gel electrophoresis photo of PCR done using *DIR 3, DIR 4* primers. M: Molecular marker with 100 bp DNA ladder, Lane 44 – Positive control, Lane 45- Negative control. Positive samples – Lane 31, 32, 33, 34, 35, 36, 37, 39, 40, 42, 43, Negative samples – 38, 41.

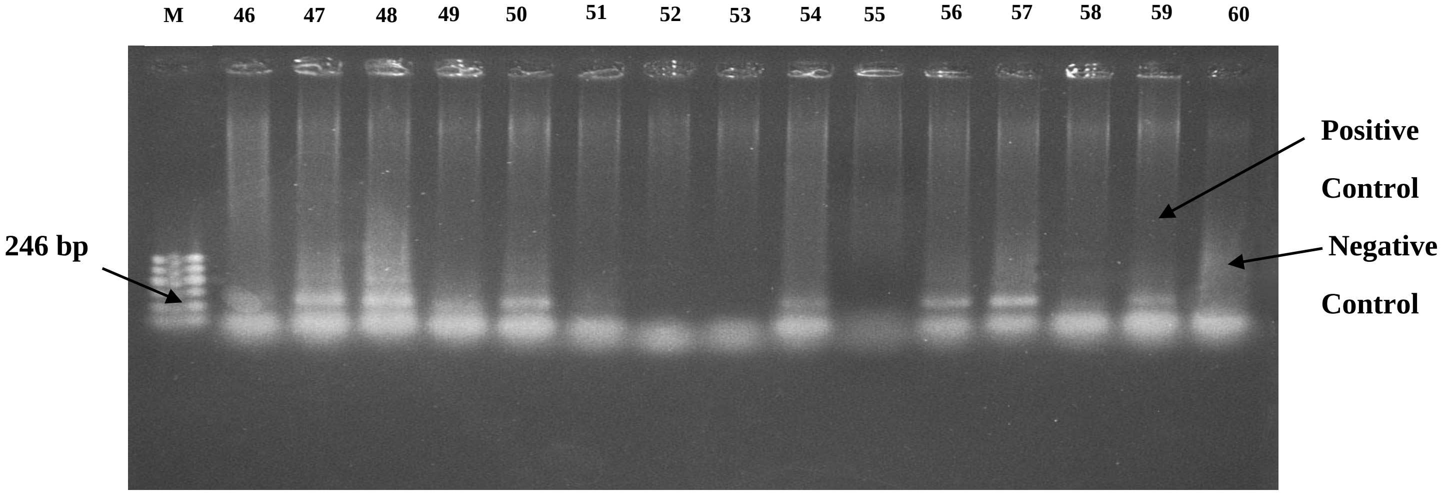

Figure 4 Gel electrophoresis photo of PCR done using *DIR 3*, *DIR 4* primers. M: Molecular marker with 100 bp DNA ladder, Lane 59 – Positive control, Lane 60 - Negative control. Positive samples – Lane 46, 47, 48, 49, 50, 54, 56, 57, 58. Negative samples –51, 52, 53, 55

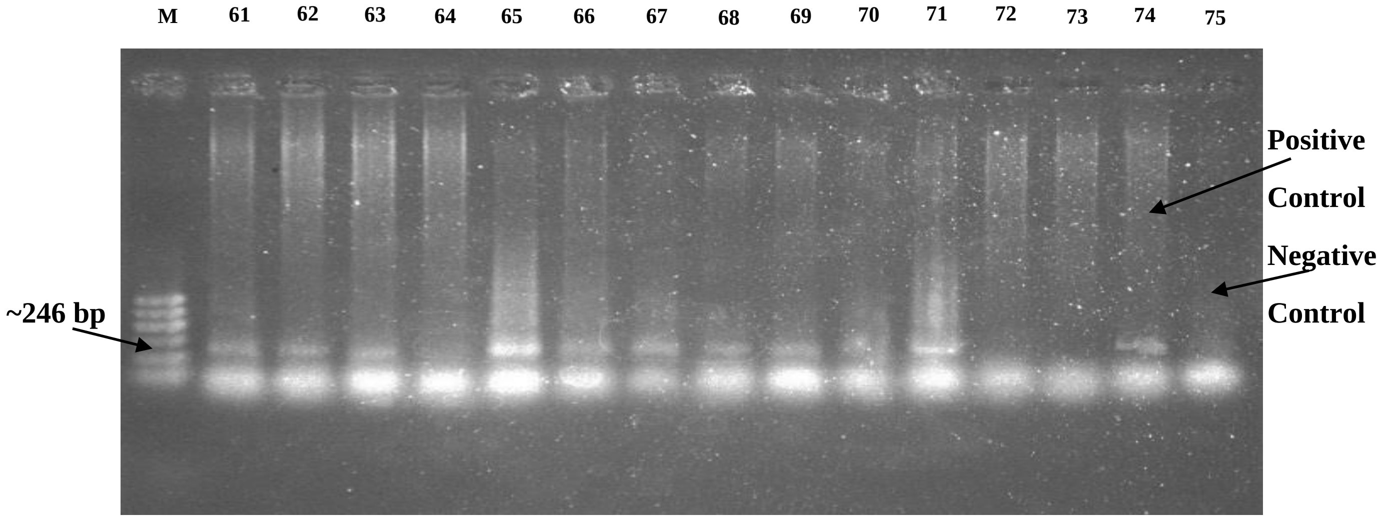

Figure 5 Gel electrophoresis photo of PCR done using *DIR 3*, *DIR 4* primers. M: Molecular marker with 100 bp DNA ladder, Lane 74 – Positive control, Lane 75- Negative control. Positive samples – 61, 62, 63, 65, 66, 67, 68, 69, 70, 71. Negative samples -64, 72, 73.

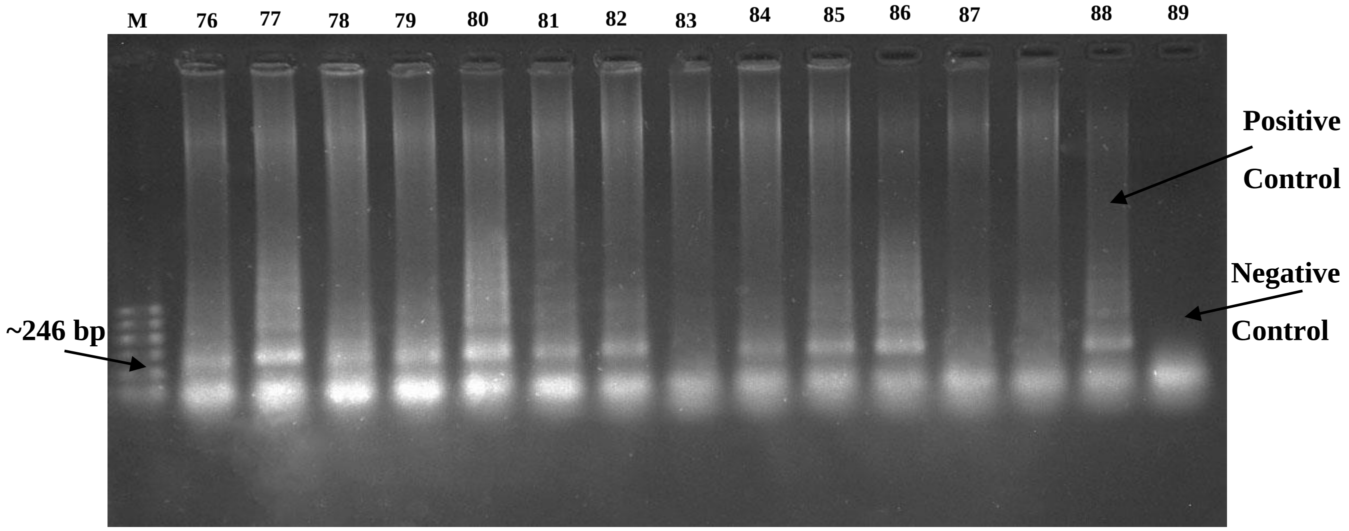

Figure 6 Gel electrophoresis photo of PCR done using *DIR 3, DIR 4* primers. M: Molecular marker with 100 bp DNA ladder, Lane 88 – Positive control, Lane 89- Negative control. Positive samples – 76, 77, 78, 79, 80, 81, 82, 84, 85, 86. Negative samples – 83, 87.

Following are the gel images for the 85 smear negative samples.

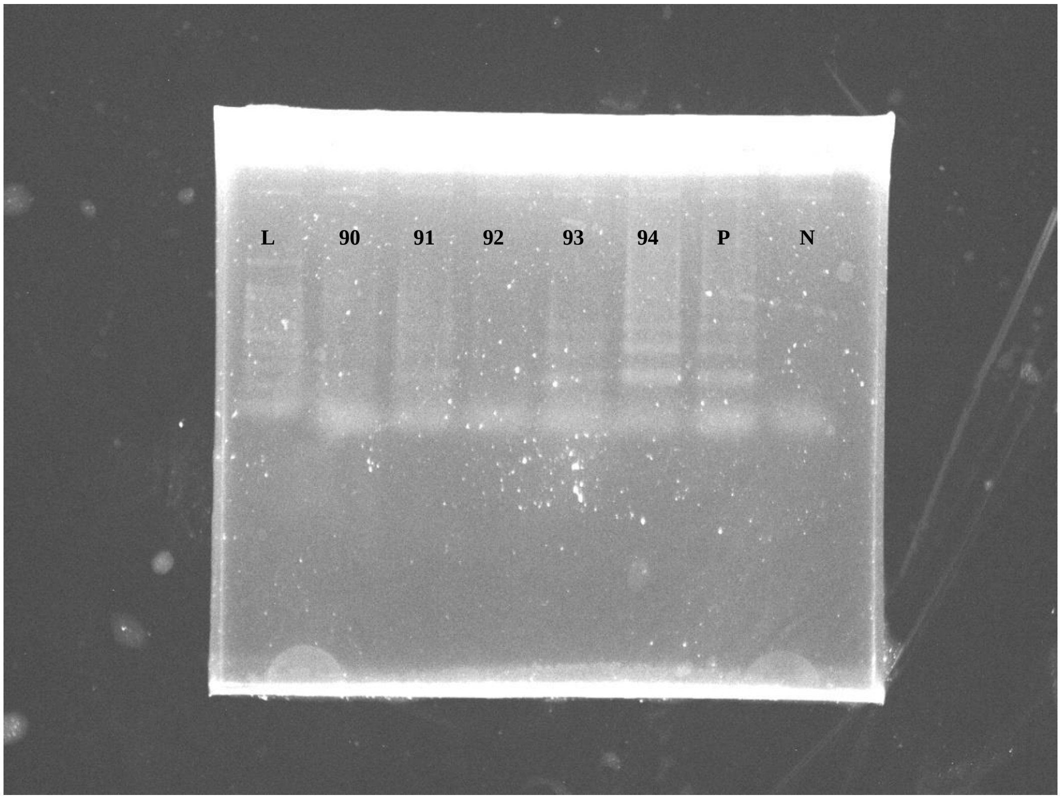

Figure 7 Gel electrophoresis photo of PCR done using *DIR 3, DIR 4* primers. M: Molecular marker with 100 bp DNA ladder, P: Positive control, N: Negative control. Positive samples –91, 93, 94. Negative samples – 90, 92.

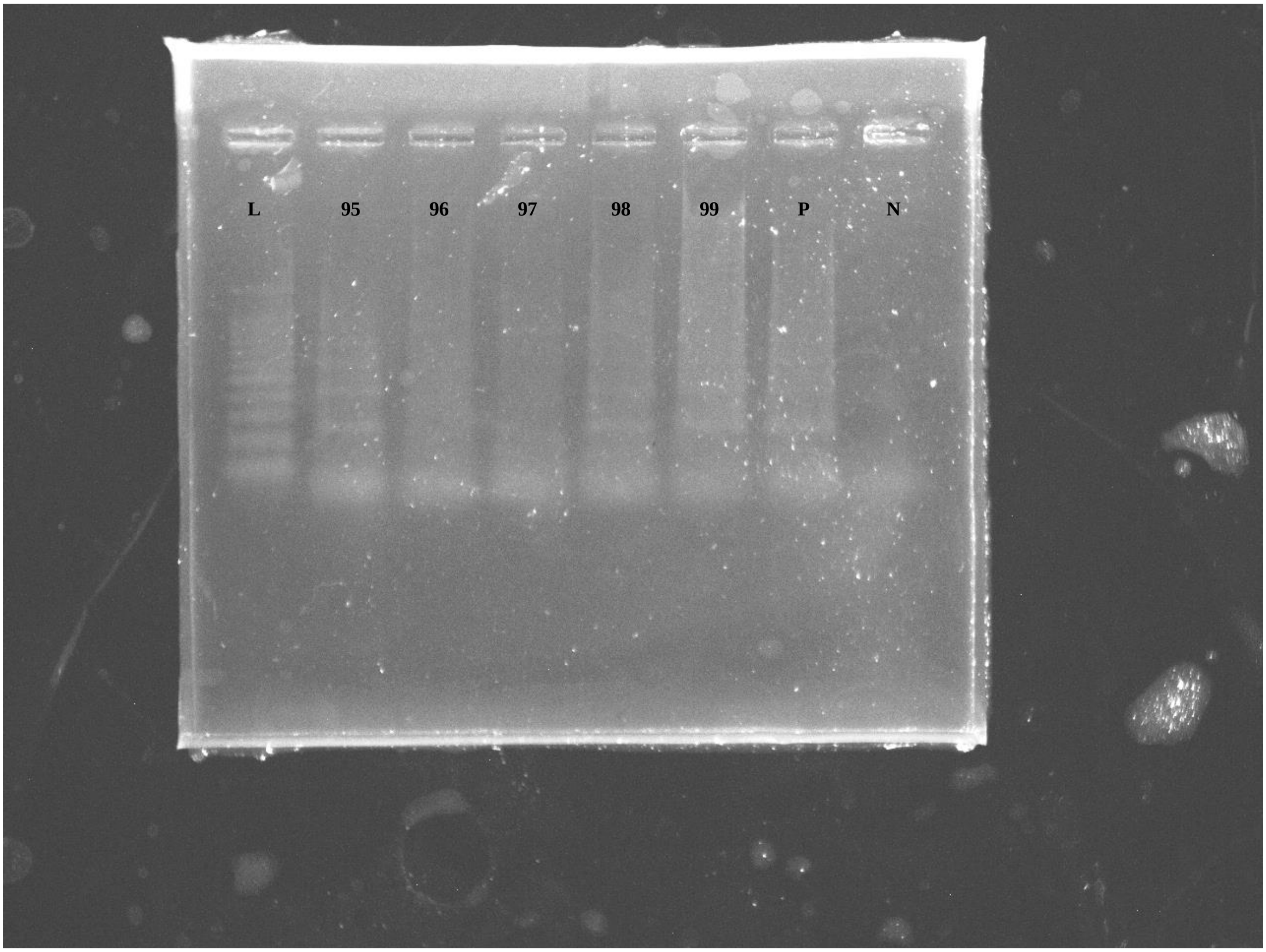

Figure 8 Gel electrophoresis photo of PCR done using *DIR 3, DIR 4* primers. M: Molecular marker with 100 bp DNA ladder, P: Positive control, N: Negative control. Positive samples –95, 98, 99. Negative samples – 96, 97.

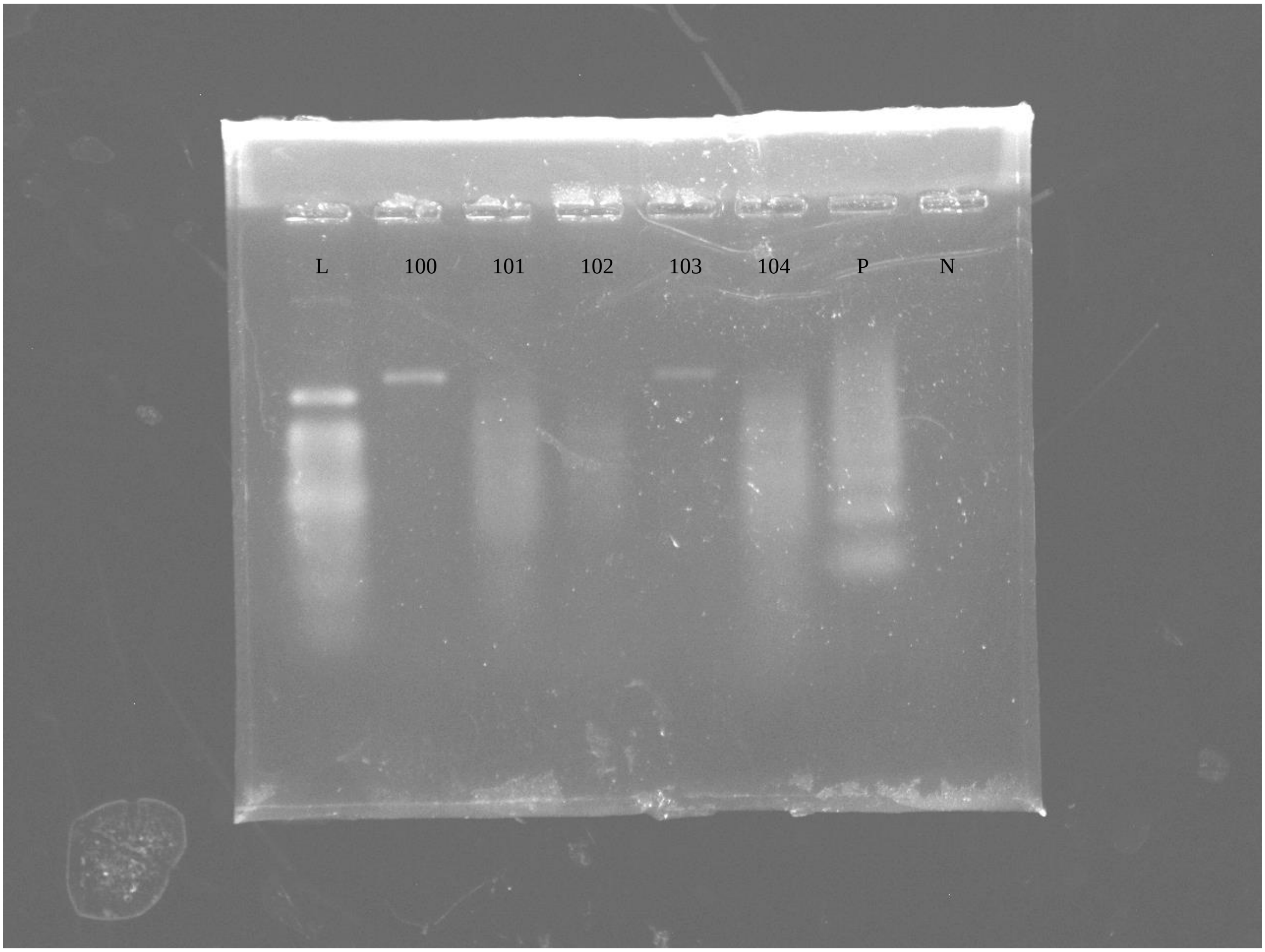

Figure 9 Gel electrophoresis photo of PCR done using *DIR 3, DIR 4* primers. M: Molecular marker with 100 bp DNA ladder, P: Positive control, N: Negative control. Negative samples – 100, 101, 102, 103, 104.

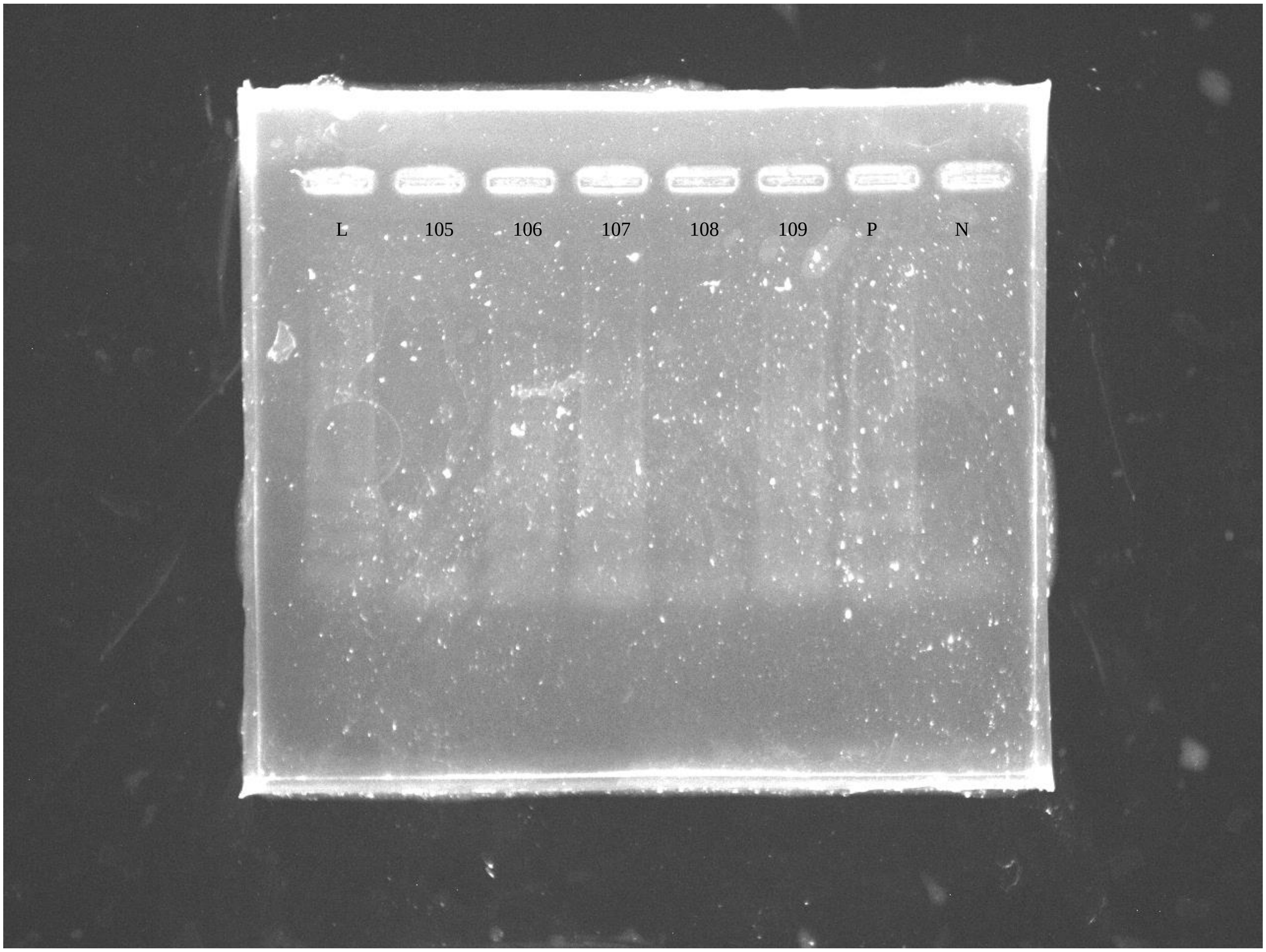

Figure 10 Gel electrophoresis photo of PCR done using *DIR 3, DIR 4* primers. M: Molecular marker with 100 bp DNA ladder, P: Positive control, N: Negative control. Negative samples – 105, 106, 107, 108, 109.

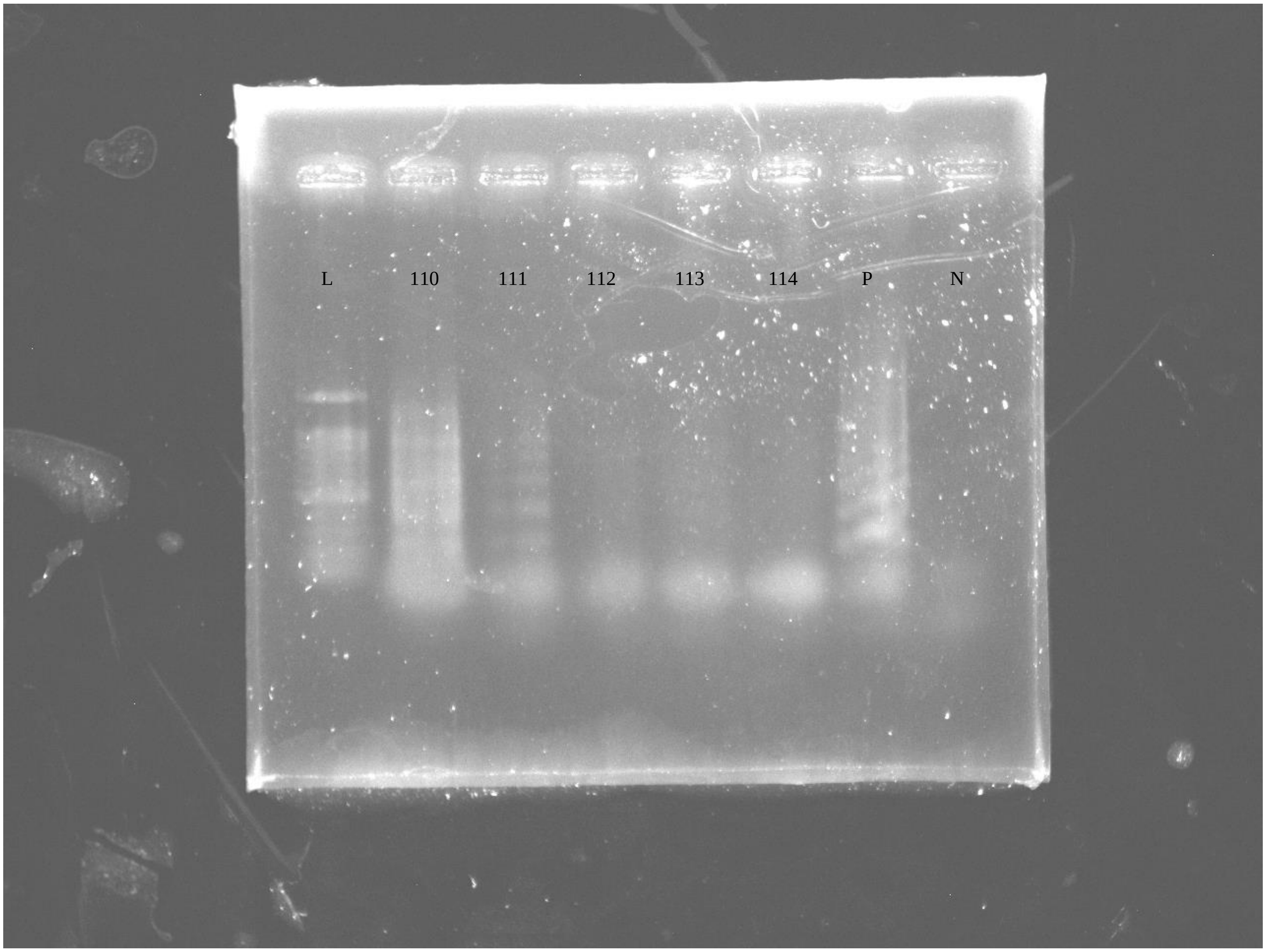

Figure 11 Gel electrophoresis photo of PCR done using *DIR 3, DIR 4* primers. M: Molecular marker with 100 bp DNA ladder, P: Positive control, N: Negative control. Positive samples – 111. Negative samples –110, 112, 113, 114.

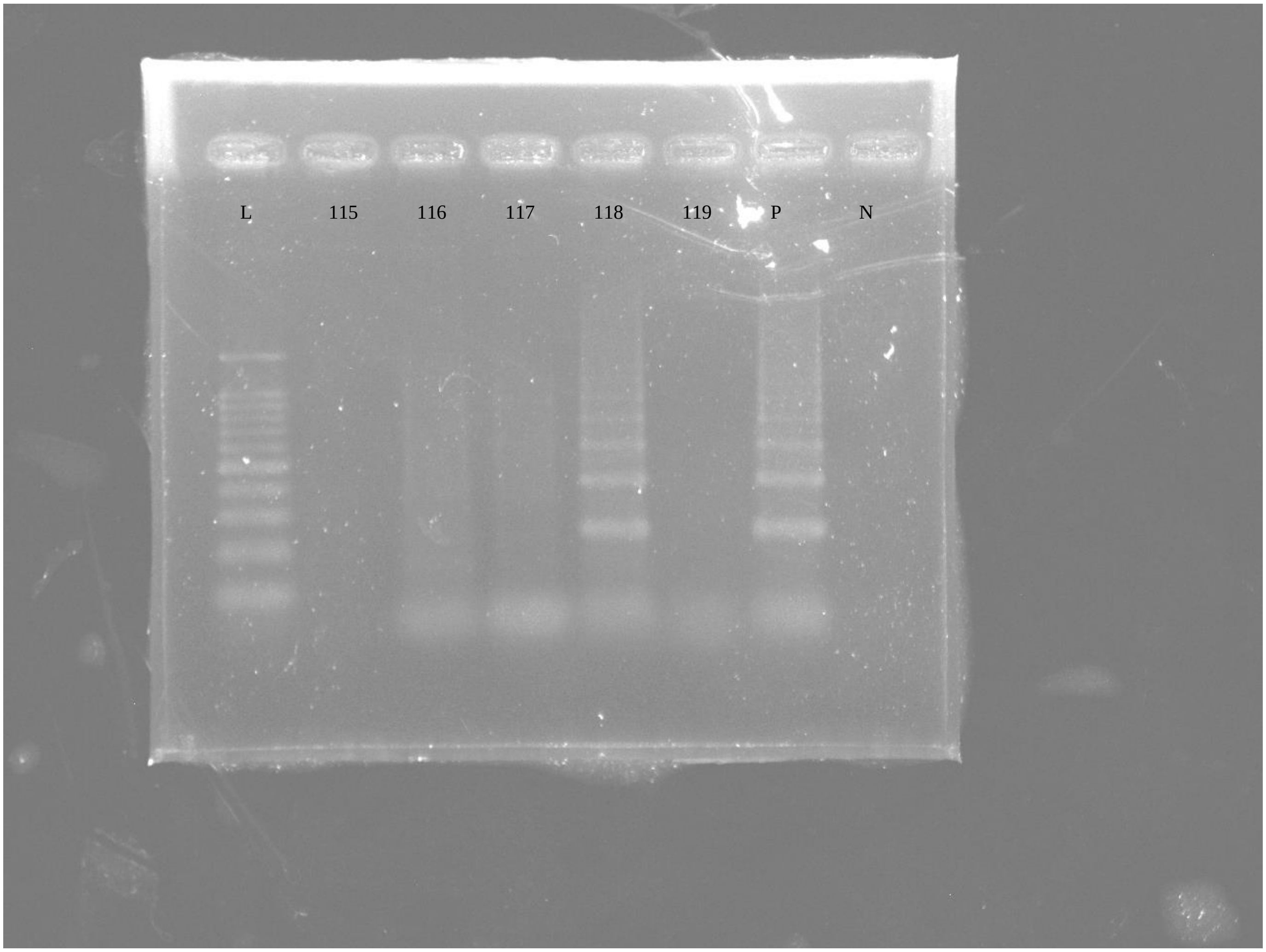

Figure 12 Gel electrophoresis photo of PCR done using *DIR 3, DIR 4* primers. M: Molecular marker with 100 bp DNA ladder, P: Positive control, N: Negative control. Positive samples – 118. Negative samples –115, 116, 117, 119.

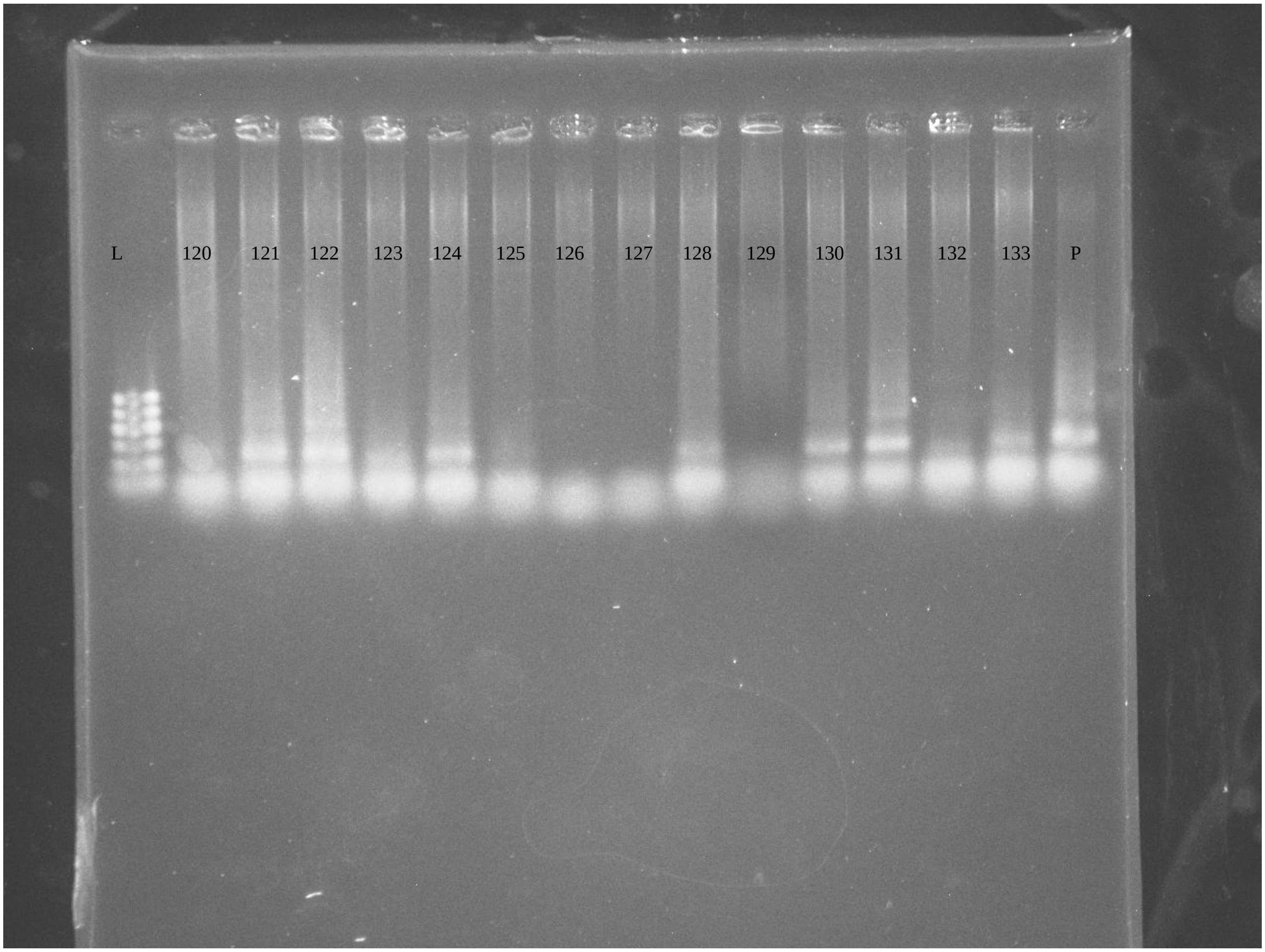

Figure 13 Gel electrophoresis photo of PCR done using *DIR 3, DIR 4* primers. M: Molecular marker with 100 bp DNA ladder, P: Positive control. Positive samples – 121, 122, 123, 124, 128, 130, 131, 132, 133. Negative samples – 125, 126, 127, 129.

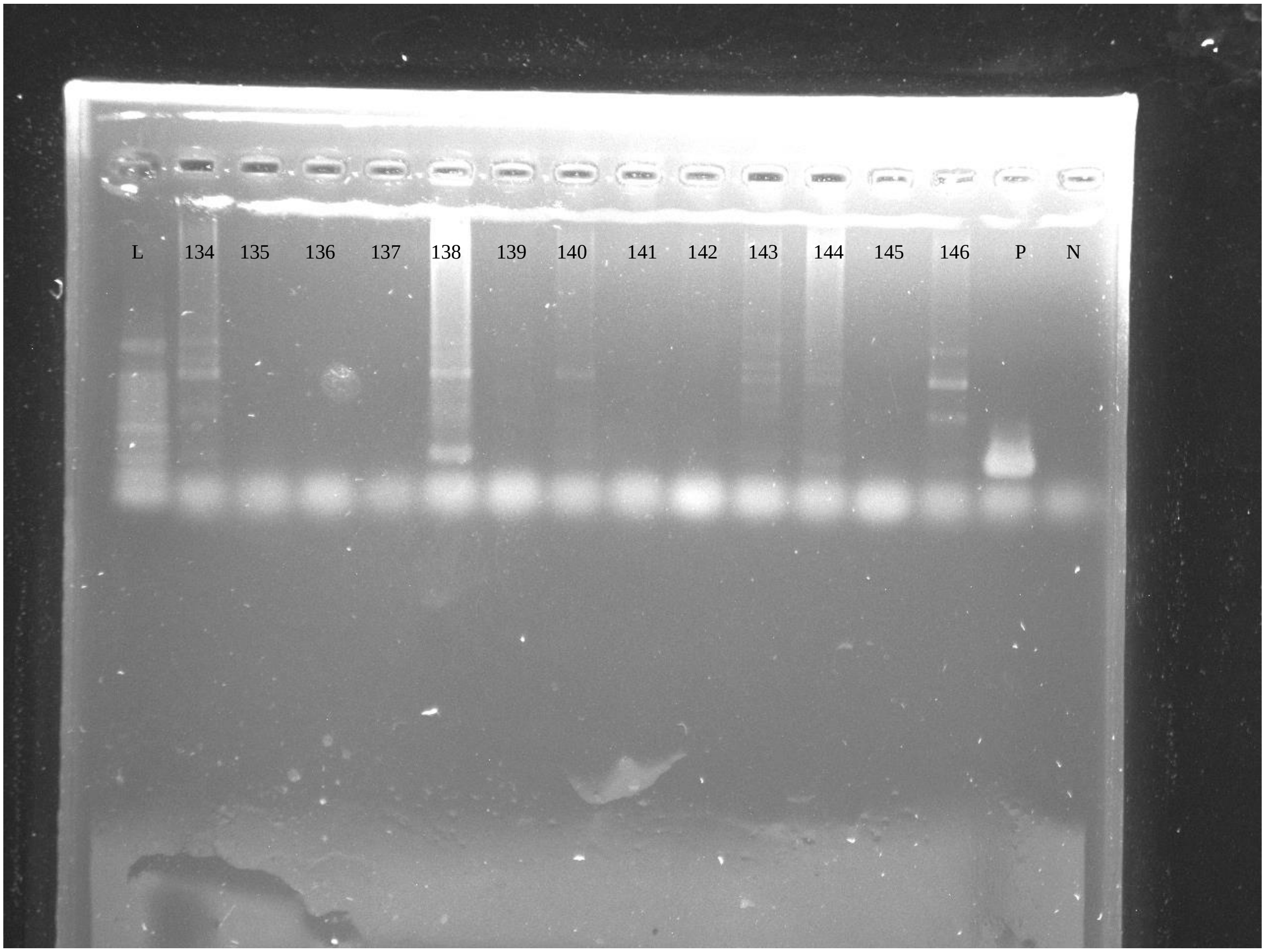

Figure 14 Gel electrophoresis photo of PCR done using *DIR 3, DIR 4* primers. M: Molecular marker with 100 bp DNA ladder, P: Positive control. Positive samples – 134, 138, 140, 143, 144, 146. Negative samples – 135, 136, 137, 139, 141, 142, 145, 146.

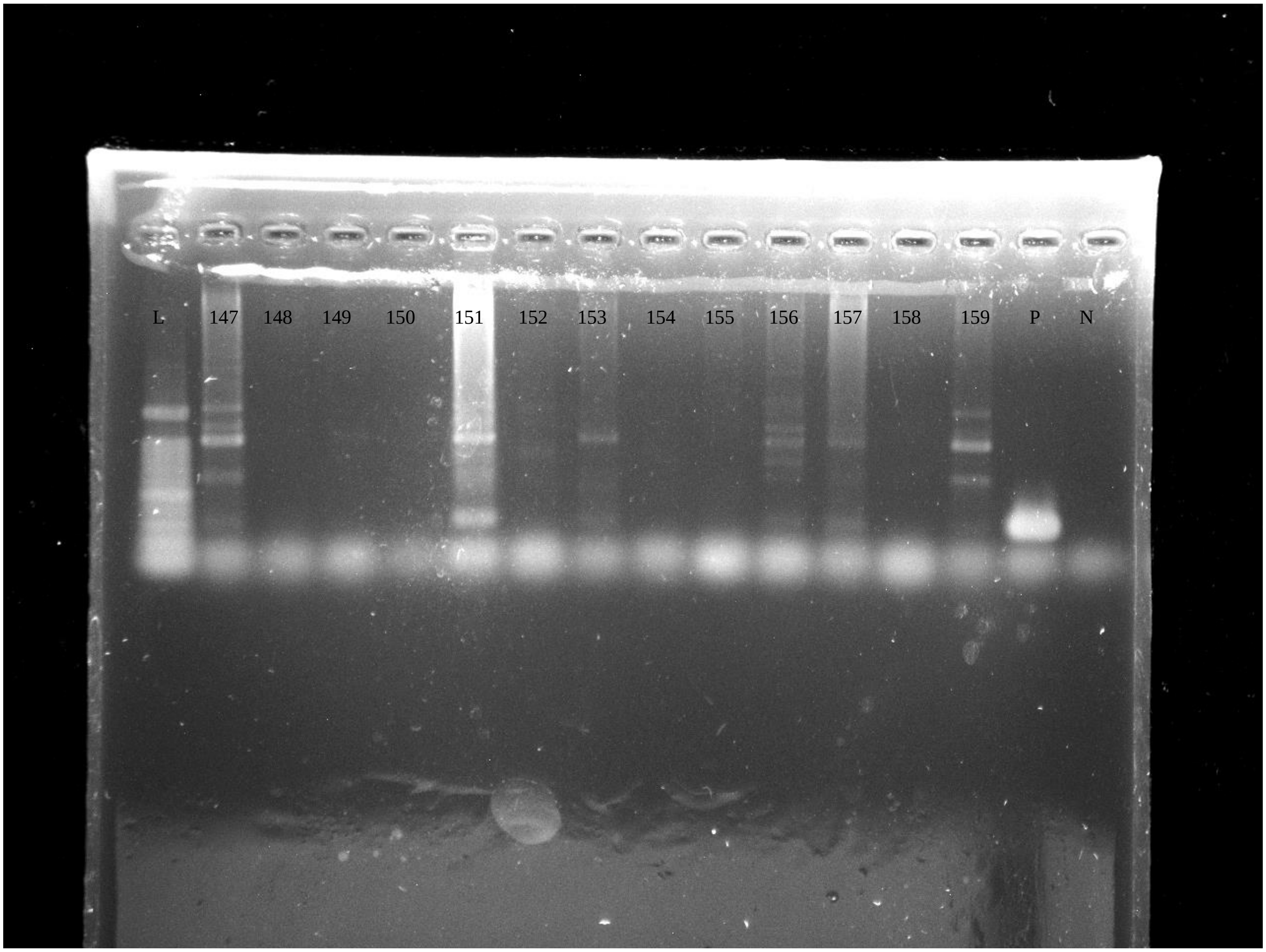

Figure 15 Gel electrophoresis photo of PCR done using *DIR 3, DIR 4* primers. M: Molecular marker with 100 bp DNA ladder, P: Positive control. Positive samples – 147, 151, 153, 156, 157, 159. Negative samples – 148, 149, 150, 152, 154, 155, 156, 158.

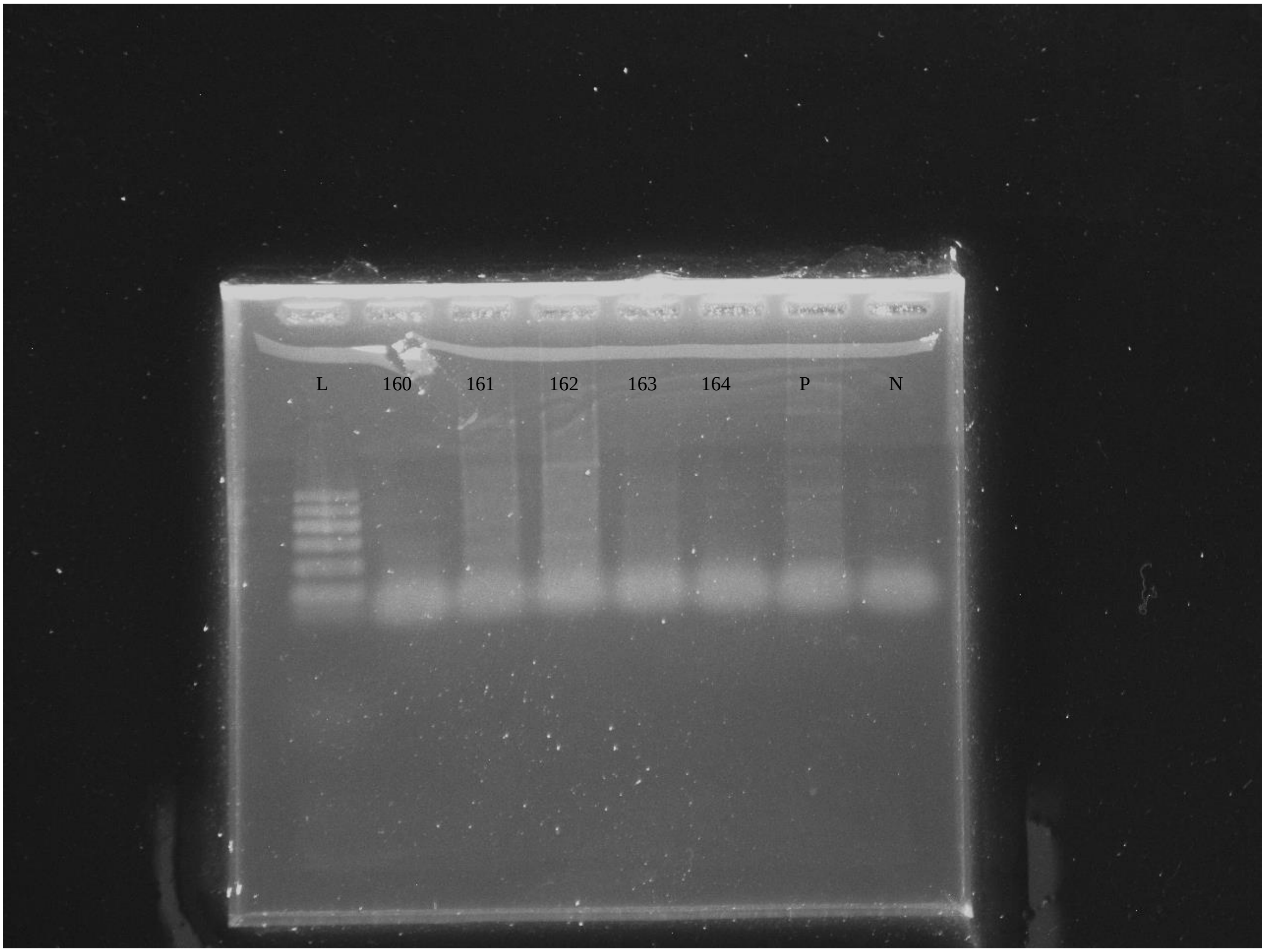

Figure 16 Gel electrophoresis photo of PCR done using *DIR 3*, *DIR 4* primers. M: Molecular marker with 100 bp DNA ladder, P: Positive control. Positive samples – 161, 162. Negative samples – 160, 163, 164
